# Primary tracheobronchial amyloidosis

**DOI:** 10.1590/0100-3984.2015.0177

**Published:** 2017

**Authors:** Pedro Paulo Teixeira e Silva Torres, Matheus Rabahi, Sebastião Alves Pinto, Karla Cristina de Morais Arantes Curado, Marcelo Fouad Rabahi

**Affiliations:** 1 Multimagem Diagnósticos, Goiânia, GO, Brazil.; 2 Pontifícia Universidade Católica de Goiás (PUC Goiás), Goiânia, GO, Brazil.; 3 Universidade Federal de Goiás (UFG), Goiânia, GO, Brazil.; 4 Hospital e Maternidade Jardim América, Goiânia, GO, Brazil.

Dear Editor,

A 58-year-old male sought treatment complaining of dyspnea on exertion, together with cough and occasional mucus secretion. He reported having been treated for asthma 14 years prior, as well as having used bronchodilators and inhaled corticosteroids, although he stated that he had experienced no asthma symptoms in childhood.

**Figure 1:**
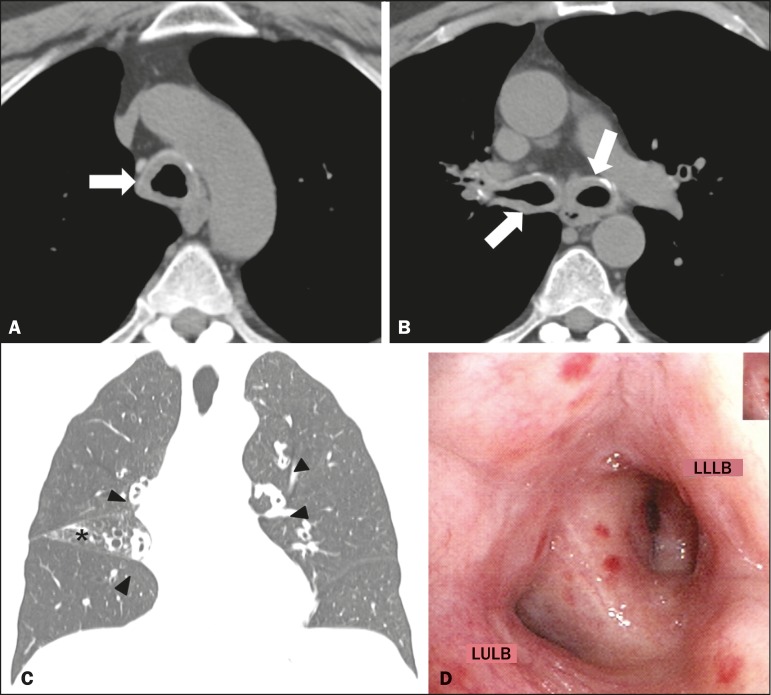
**A,B:** Axial CT scan of the chest (mediastinal window), without contrast administration, at the level of the proximal segment of the trachea (**A**) and below the carina (**B**), showing significant concentric thickening of the wall and small calcifications in the trachea and bronchi (arrows). **C:** Coronal CT scan of the chest (lung window) showing homogeneous wall thickening affecting the segmental and subsegmental bronchi (arrowheads). Signs of volumetric loss in the middle lobe (asterisk) due to narrowing of the lumen of respective lobar bronchus (not shown). **D:** Bronchoscopic image showing narrowing of the bronchial lumen by concentric, diffuse infiltration by grayish-yellow mucous, resulting in enlargement of the secondary carina. LULB, left upper lobe bronchus; LLLB, left lower lobe bronchus.

Computed tomography (CT) was performed (Figures 1A, 1B and 1C), after which the patient was submitted to bronchoscopy (Figure 1D) with biopsy. The CT showed concentric thickening of the walls of the trachea, as well as of those of the main, lobar, segmental, and subsegmental bronchi, with small calcifications. The bronchoscopy showed diffuse, concentric infiltration of the mucosa, the infiltrate having a grayish-yellow appearance. The histopathological study showed deposition of amorphous material, whose characteristics were compatible with amyloid deposits.

Amyloidosis encompasses a set of diseases characterized by deposition and abnormal accumulation of protein material in organs and tissues^(1)^. Depending on the anatomical distribution, amyloidosis can be classified as systemic (involving multiple organs) or localized (involving a single organ). In the biochemical classification, based on the type of fibrillar component in amyloid deposits, there are innumerable subtypes. In the vast majority of cases, light-chain amyloid fibrils and serum amyloid A are identified^(1)^.

In the thoracic compartment, amyloidosis typically affects the heart but can also involve the pulmonary parenchyma, pleura, lymph node chains, tracheobronchial tree, and other sites^(1,2)^. Pulmonary involvement is rare, reported as tracheobronchial, diffuse/alveolar-septal, or nodular manifestations, the first being the most common^(2-4)^.

The tracheobronchial manifestation of amyloidosis is characterized by the deposition of amyloid material in the trachea and main bronchi, resulting in thickening of the walls, narrowing of the lumina, and consequent airway obstruction, as well as consolidations, atelectasis, pulmonary hyperinflation, and bronchiectasis^(3)^.

Clinically, amyloidosis-related tracheobronchial impairment can be asymptomatic or can manifest as dyspnea, wheezing, hemoptysis, cough, or recurrent pneumonia^(4,5)^. The symptoms can be similar to those of bronchial diseases that are more common, including bronchial asthma^(5)^.

Chest CT has been shown to be the imaging exam of choice for the evaluation of thoracic diseases^(6-9)^, as well as for that of diseases of the tracheobronchial tree^(10-12)^. In individuals with amyloidosis, a CT scan can reveal smooth or irregular/nodular thickening of the tracheal wall and bronchi, which can be accompanied by calcified nodules in the submucosa^(4)^. The differential diagnoses of diffuse tracheobronchial diseases include vasculitis (Wegener's granulomatosis), tracheobronchial papillomatosis, infectious involvement (rhinoscleroma, caused by infection with Klebsiella rhinoscleromatis), tracheopathia osteochondroplastica, and relapsing polychondritis^(13)^. Unlike tracheal involvement in tracheopathia osteochondroplastica or relapsing polychondritis, tracheobronchial amyloidosis involves the posterior membranous wall of the trachea^(4,13)^.

In individuals with amyloidosis, bronchoscopy usually shows thickening of the walls of the trachea and bronchi, with flat, multifocal, grayish-yellow plaques in the trachea and bronchi. In rare cases, amyloid pseudotumors can be seen^(5,13)^. Histopathological findings of the disease include amyloid thickening of the submucosa, in nodular masses or laminae, showing apple-green birefringence after staining with Congo red^(14)^. There is also a reduction in the number of submucosal glands, together with calcifications and foci of bone metaplasia in the upper airways^(14)^.

In patients suspected of having bronchial asthma who present with atypical symptoms and respond poorly to clinical treatment, various differential diagnoses should be considered^(15)^. The patient in question was initially diagnosed with asthma but did not respond to treatment, and the definitive diagnosis of primary tracheobronchial amyloidosis was made after a directed follow-up assessment. We can conclude that, albeit rare, tracheobronchial amyloidosis should be considered in such patients.
